# High-Efficiency Iron Extraction from Low-Grade Siderite via a Conveyor Bed Magnetization Roasting–Magnetic Separation Process: Kinetics Research and Applications

**DOI:** 10.3390/ma15186260

**Published:** 2022-09-08

**Authors:** Shaowu Jiu, Bo Zhao, Chao Yang, Yanxin Chen, Fuan Cheng

**Affiliations:** College of Materials Science and Engineering, Xi’an University of Architecture and Technology, Xi’an 710055, China; jiushaowu@xauat.edu.cn (S.J.); zhaobo@xauat.edu.cn (B.Z.); ycwork@xauat.edu.cn (C.Y.)

**Keywords:** siderite, magnetization roasting, in-situ infrared, kinetics, conveyor bed, magnetic separation

## Abstract

Upgrading and utilizing low-grade iron ore is of great practical importance to improve the strategic security of the iron ore resource supply. In this study, a thermal analysis–infrared (IR) analysis–in-situ IR method was used to investigate the reaction mechanism and kinetics of Daxigou siderite. Experiments were conducted using a conveyor bed magnetization roasting process (CBMRP) to investigate the magnetization of siderite. Multi-stage magnetic separation processes were adopted to extract magnetite. The results show that simultaneously the iron carbonate in siderite decomposes, and magnetite is formed between 364 °C and 590 °C under both inert and reducing atmospheres. The activation energy of the magnetization roasting reaction is 106.1 kJ/mol, consistent with a random nucleation and growth reaction mechanism. Magnetization roasting at 750–780 °C for approximately 3.5 s in the CBMRP results in a magnetic conversion rate of >0.99 of the iron minerals in the siderite. A beneficiation process of one roughing, one sweeping, and three cleaning processes was adopted. A dissociation particle size of −400 mesh accounting for 94.78%, a concentrate iron grade of 62.8 wt.%, and a recovery of 68.83% can be obtained. Overall, a theoretical and experimental basis is presented for the comprehensive utilization of low-grade siderite.

## 1. Introduction

Siderite is a low-grade, difficult-to-sort iron ore comprising iron carbonate (FeCO_3_) as its main component [1]. The known iron ore reserves in China may be as high as 1.8 billion tons, representing waste that is effectively unutilized at present [2]. Siderite resources in China are mainly located in Yunnan, Shaanxi, Gansu, Qinghai, and Xinjiang, all of which have reserves of >100 million tons of ilmenite. The Daxigou siderite in Shangluo City, Shaanxi Province, is the largest siderite deposit in China, with a reserve of >300 million tons. With the continuous and rapid development of the iron and steel industry in China, high-quality iron ore resources are being depleted. Therefore, developing a comprehensive technology for utilizing low-grade iron ore resources is of great practical significance for the sustainable development of the iron and steel industry [1,3,4].

When siderite is roasted under an inert or reducing atmosphere, its FeCO_3_ component decomposes and gradually converts to magnetite [2,3,4], which can then be magnetically separated from non-magnetic veinstone minerals [5,6,7]. Magnetization roasting and magnetic separation are thus two key technologies employed in the comprehensive utilization of siderite [8,9,10,11]. To date, rotary kiln, fluidized bed, and conveyor bed methods have been developed and used in China as methods for the magnetization roasting of siderite [12,13]. The conveyor bed magnetization roasting process (CBMRP, also known as suspension roasting) is a process that has been recently developed for the magnetization roasting of siderite employing micron-sized powder as a raw material that has been fully magnetized via roasting at 600–800 °C for 3–10 s under a reducing atmosphere. CBMRP is a simple process with high reaction efficiency, and large processing capacity, and the waste heat of the reaction can be recovered and the exhaust gas can be easily purified, which has significant technical advantages compared with the rotary kiln process [8,14]. To date, a large number of conveyor bed roasting process pilot tests have been completed and have been employed in the construction of production lines [15,16,17]. As the magnetization roasting time of the conveyor bed is very short, the magnetization roasting of siderite is very sensitive to the influence of the atmosphere, temperature, and time. Therefore, an accurate model of the reaction kinetics is required to provide a basis for process design and production control.

Numerous studies have shown that the magnetization roasting of iron-based minerals is influenced by the calcination method, reaction atmosphere, and parameters of the calcination conditions (temperature, time, particle size, etc.), and their reaction mechanism and kinetics are very complex [2,3,8,10,11,12]. In the magnetization roasting of siderite, not only the decomposition of FeCO_3_ occurs but also the phase transformation of iron minerals [2,3,4]. To date, most of the studies on iron mineral transformations have employed intermittent sampling and analysis methods, which are appropriate for slow calcination processes involving rotary kilns and fluidized beds, but there are limitations in the analysis of iron mineral transformations under the fast conditions of CBMRP. In-situ infrared (IR) analysis is a technology that is used in the online detection of materials structures and is widely used in the reaction characterization of organic compounds [18,19,20]. However, conventional in-situ IR analysis can only be used at temperatures <500 °C and cannot be introduced into a reducing reaction atmosphere, which limits its application in the reduction reaction of metal minerals. In this study, a specially-modified online in-situ IR analyzer with programmed temperature control and use under a reducing atmosphere in the range of 25–750 °C was developed. A specially-tailored potassium bromide (KBr) glass was used to extend the wavenumber range of an IR absorption spectrometer to a range of 4000–400 cm^−1^, enabling the detection of the IR absorption characteristics of iron minerals in the low-wavenumber region. Using this spectrometer solves the issues associated with detecting the transformation process of iron minerals that occurs during the magnetization roasting of siderite, thus providing a basis for the study of its reaction mechanism and kinetics. To date, there is still a lack of systematic studies on the application of magnetization reaction kinetics of siderite to the design and control practices of industrial production lines. In addition, studies on the reaction characteristics of siderite in CBMRP and the beneficiation process of low-grade siderite roasted ores are also necessary.

In this study, Daxigou siderite was used as raw material. The reaction process, iron mineral transformation, and reaction kinetics of siderite were characterized by combined thermal analysis–IR analysis and online in-situ IR analysis. Kinetic equations were used to predict the magnetization reactions of siderite at different temperatures, and CBMRP pilot tests were performed. The effect of magnetization was investigated by conducting a mineral phase analysis of the roasted ore in terms of magnetic conversion rate, and the optimal magnetization roasting parameters were determined. A multi-stage magnetic separation process was used to analyze the beneficiation of roasted ore produced by CBMRP, and the beneficiation effect was characterized according to the grade and recovery of iron concentrate. A systematic approach is established in this study in terms of the reaction mechanism, kinetics, magnetization roasting process system, and beneficiation process of siderite, which serves as a reference for the development, process design, and production control of conveyor bed magnetization technology.

## 2. Materials and Methods

### 2.1. Raw Materials

Daxigou siderite was used as raw material, procured from Shangluo City, Shaanxi Province, China. Siderite is a sedimentary metamorphic iron ore, with an iron grade of 16–23%; a typical low-grade type of siderite that is difficult to concentrate. A total of 32 tons of raw material of siderite was collected, which was the same batch of raw material used for production by the Daxigou Mining Company of the Shaanxi Long Steel Group. The raw ore was crushed and ground into a powder using a vertical mill, and 98.25% of the final particle size passed through a −200 mesh standard sieve (*d*_50_ = 42.85 μm). The finished material after grinding was thoroughly mixed and 50 kg of raw material was collected for chemical composition analysis using multi-point sampling. The rest of the powder was loaded into a silo as a raw material for CBMRP testing.

**Figure 1:**
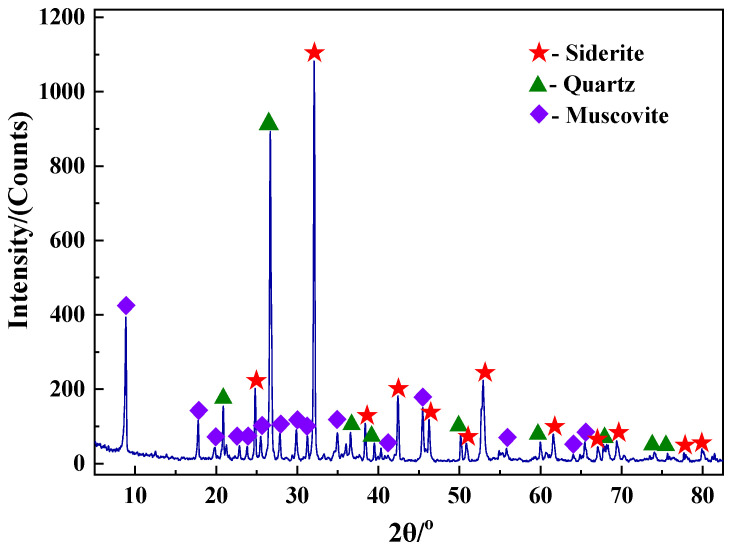
XRD pattern of siderite.

The mineral phase analysis of siderite was conducted by X-ray diffractometry (XRD, D/MAX-2200, RIKEN, Wako City, Japan) using a Cu Kα radiation source, tube voltage of 45 kV, tube current of 40 mA, and scanning was conducted over a 2*θ* range of 5–85° with a scanning speed of 5°/min. The XRD pattern of siderite is shown in Figure 1.

The chemical elements in siderite were analyzed by X-ray fluorescence spectroscopy (XRF, S4PIONEER, Bruker, Germany), using X-ray tube parameters of 4.2 kW, 60 kV (max), and 140 mA (max). The quantitative method without a standard sample was adopted. The grade of iron of siderite was analyzed using a chemical elemental analysis method, the results of which are shown in Table 1.

Figure 1 and Table 1 show that the major minerals in the raw material are siderite, quartz, and muscovite, of which FeCO_3_ is the major iron mineral component. In addition, siderite also contains minerals of Mn, Mg, S, and other elements. The average iron grade of Daxigou siderite is 21.42 wt.%, which makes it a low-grade iron ore.

### 2.2. Reaction Mechanism Analysis

A combined system comprising a thermogravimetric analysis-differential scanning calorimetry (TGA-DSC) simultaneous thermal analyzer (409PC, NETZSCH, Bavaria, Germany) and an IR spectrometer (FTIR-7600, Bruker, Karlsruhe, Germany) was used to detect the mass changes and gas products of siderite during the magnetization roasting process. The experimental parameters were as follows. The sample mass was 10.0 ± 0.5 mg, the atmosphere was high purity Ar gas, the flow rate was 45.0 mL/min, the heating rates were 10 °C /min, 15 °C /min, 20 °C /min, and 25 °C /min, and the wavenumber range of the FTIR detector was 4000–400 cm^−1^. A high-temperature and high-pressure diffuse reflectance in-situ analyzer (Specac, Orpington, UK) combined with an IR spectrometer (Spectrum 3, PerkinElmer, Waltham, MA, USA) were used to examine the changes in the physical phase structure of siderite during the magnetization roasting process. The parameters of the online in-situ IR analysis were a temperature range of 25–750 °C, a heating rate of 15 °C/min, a wavenumber range of 4000–400 cm^−1^, KBr glass as the medium, and a mixed high purity Ar and CO (Ar 99% + CO 1%) atmosphere controlled at a flow rate of 65 mL/min.

### 2.3. Kinetics Analysis Method

The thermoanalytical kinetic equation for the reaction of solid substances is as follows [21,22]:

d*α/*d*T* = *A*exp[−*E*/(R*T*)]*f*(*α*) (1)

where *α* is the reaction conversion rate; *A* is the finger front factor of the kinetic equation, s^−1^; *E* is the reaction activation energy, kJ/mol; R is the gas constant with a value of 8.314 J/(mol·K); *T* is the temperature, K; *f*(*α*) is the differential form of the mechanism function; and d*α*/d*T* is reaction rate.

Equation (1) is the kinetic equation for thermal analysis in differential form. The kinetic equation in integral form is given by Equation (2):

G(*α*) = *A*exp(−*E*/R*T*)*t* (2)

where *t* is the time, s, and G(*α*) is the integral form of the mechanism function. The G(*α*) functions commonly used for solid-phase reactions were taken from the literature [23].

The formula for calculating the reaction conversion *α* when using thermogravimetric data is [23]

*α* = (*m*_0_ − *m_i_*)/(*m*_0_ − *m*_∞_) (3)

where *m*_0_, *m_i_*, and *m*_∞_ are the masses of the samples at the beginning, during, and at the end of the reaction, respectively.

*E* and *A* were calculated and G(*α*) was determined by fitting the *α* − *T* data using the Kissinger method and the general integration method [24,25]. The Kissinger equation is as follows [24]:

(4) $$\ln\left(\frac{\beta_{i}}{T_{\mathrm{pi}}{}^{2}}\right)=\ln\frac{AR}{E}-\frac{E}{R}\frac{1}{T_{\mathrm{pi}}},\;i=1,2,3,4$$

where *β_i_* is the heating rate, °C/min, and *T_pi_* is the peak temperature in the DTG curve, K.

The general integral method is as follows [25]:

(5) $$\ln\left[\frac{G(\alpha )}{T^{2}}\right]=\ln\left\{\frac{AR}{\beta E}\left(1-\frac{2RT}{E}\right)\right\}-\frac{E}{RT}$$

For a suitable G(*α*), ln[G(*α*)/*T*^2^] − 1/*T* in the above equation is a linear relationship, from which the value of *E* is obtained from the slope of the line, and the value of *A* is obtained from the intercept. The kinetic equations of the magnetization roasting of siderite were obtained by substituting *E, A*, and G(*α*) into Equation (2).

### 2.4. Conveyor Bed Magnetization Roasting Pilot Test System

**Figure 2:**
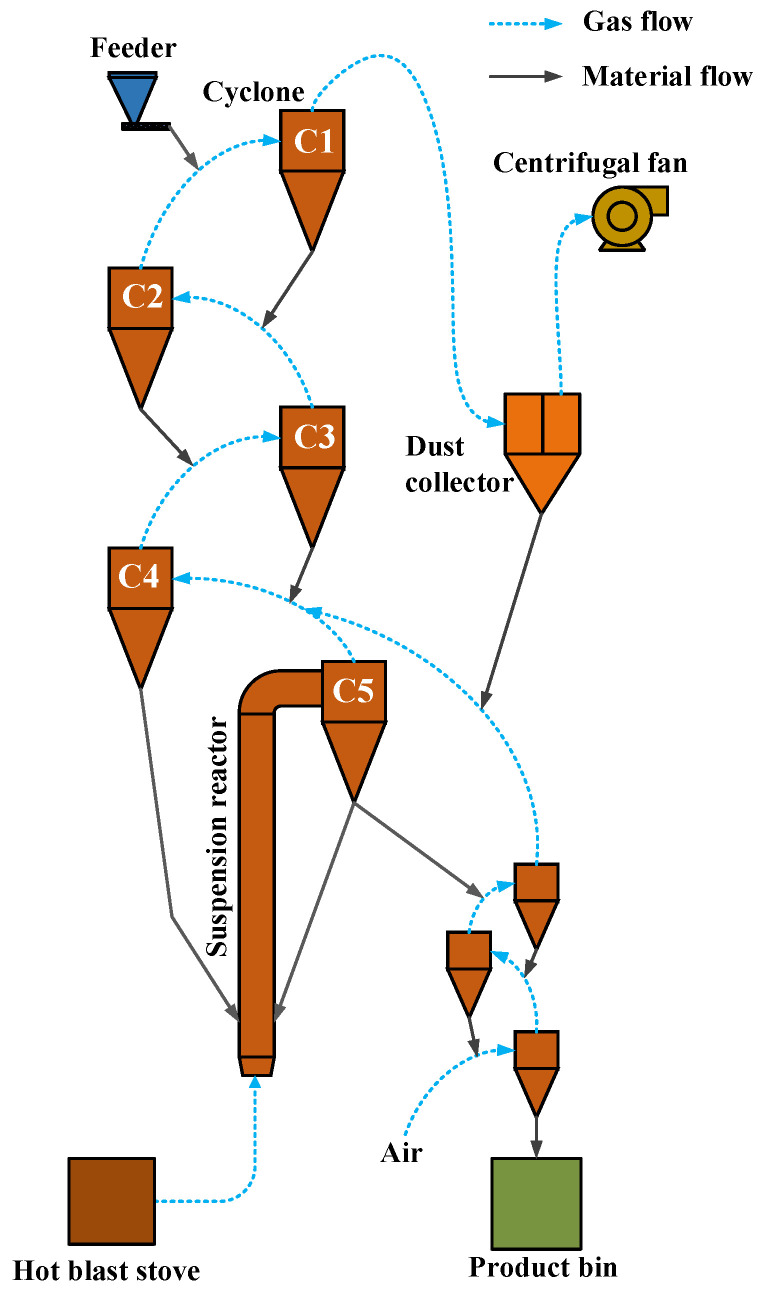
Conveyor bed magnetization roasting process system.

The conveyor bed magnetization roasting pilot test system is shown in Figure 2.

In the pilot tests, the controlled roasting temperature was in the range of 600–780 °C (middle furnace temperature), the CO content in the C5 exit gas was 0.8–1.5%, the residence time of the material in the suspension reactor was around 3.5 s, the charge was around 800 kg per hour, and the continuous operation time was 72 h.

### 2.5. Characterization of the Roasted Ores

XRD analysis was performed on the roasted ore to determine its iron mineral composition. The iron content in various minerals was analyzed by chemical titration analysis. The magnetic conversion rate *η* was calculated to characterize the magnetization roasting effect:

*η* = *Q*_m_/*Q*_Total_ × 100% (6)

where *Q*_m_ is the mass of iron elements in magnetite and *Q*_Total_ is the total mass of iron elements that can be magnetized. As iron in silicates cannot be magnetized, *Q*_Total_ is equal to the mass of iron in the roasted ore with the mass of iron in the silicates subtracted. The magnetized roasted ore was subjected to backscattered electron (BSE) imaging and energy-dispersive spectroscopy (EDS) at 750 °C to determine its reasonable dissociation particle size and beneficiation process.

### 2.6. Magnetic Separation Analysis

**Figure 3:**
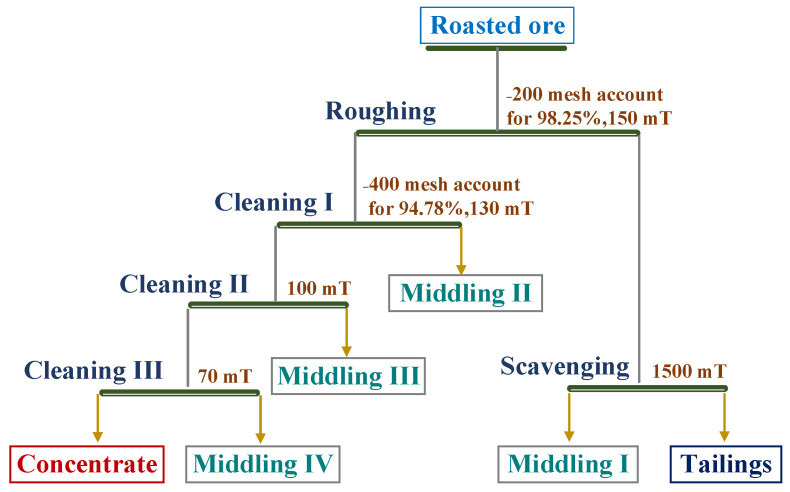
Flow chart of the magnetic separation process.

The magnetic separation involved a multi-stage magnetic separation process of one roughing, one sweeping, and three cleaning stages. The beneficiation parameters and process are shown in Figure 3.

XRD analysis and chemical titration analysis were conducted on the middling, concentrate, and tailings of the beneficiation. The composition of the iron minerals was identified by XRD analysis, and the iron grade was analyzed by chemical titration. The yield and recovery were also calculated.

## 3. Results and Discussion

### 3.1. Reaction Mechanism Analysis

**Figure 4:**
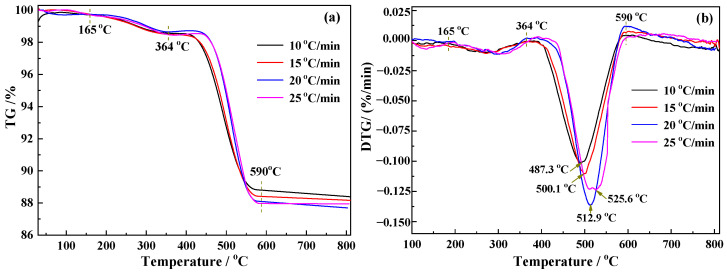
(**a**) TGA and (**b**) DTG curves for the roasting of siderite.

The thermogravimetric analysis (TGA) and differential thermogravimetry (DTG) profiles of the thermal analysis of siderite are shown in Figure 4a,b.

As shown in Figure 4a,b, there are three stages of mass loss of siderite that occur during the magnetization roasting process. The temperature range of the first stage is 90–165 °C with an average mass loss of 0.52 wt.%. The second stage is in the range of 165–364 °C with an average mass loss of 0.98 wt.%. The third stage is the main reaction stage with a temperature range of 364–590 °C, producing an average mass loss of 10.1 wt.%.

**Figure 5:**
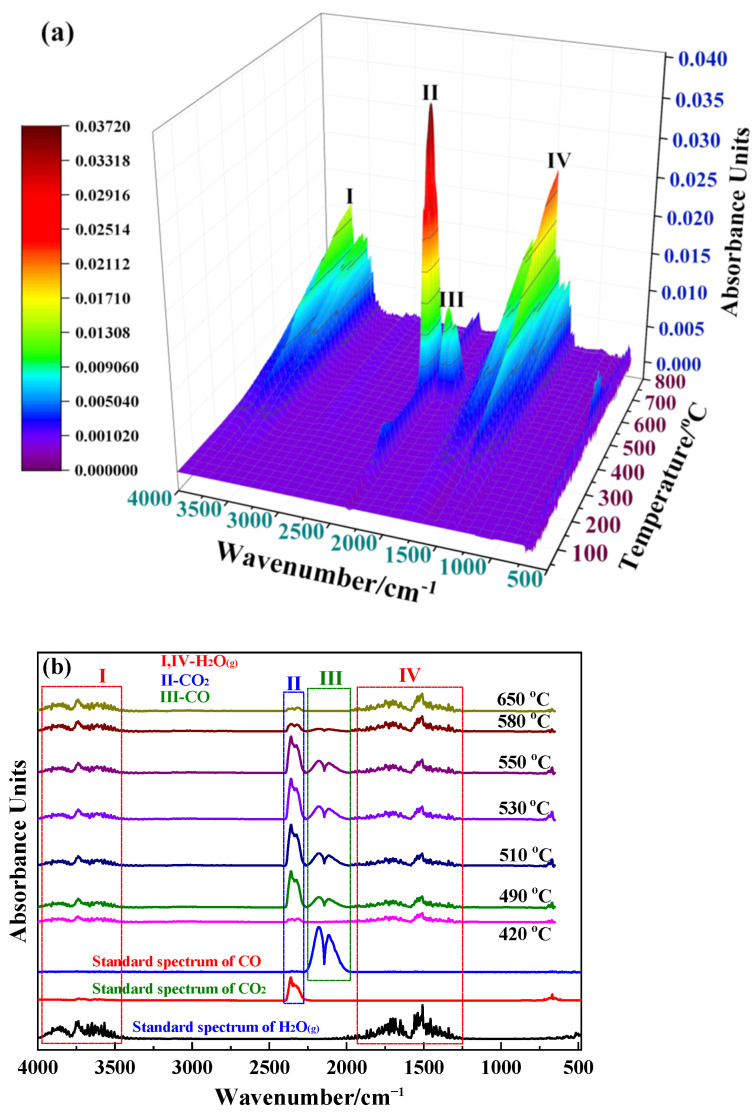
(**a**) 3D and (**b**) 2D FTIR spectra of the gaseous products.

The reaction processes were analyzed from the IR data of the gaseous products released from siderite during magnetization roasting. The three-dimensional (3D) IR absorption spectra of the gaseous products recorded at a heating rate of 15 °C/min are shown in Figure 5a. The two-dimensional (2D) infrared absorption spectra resolved from Figure 5a are shown in Figure 5b.

As shown in Figure 5b, the absorption bands of three functional groups, hydroxyl, and two carbon-oxygen groups, are mainly detected in the gaseous products released from the magnetization roasting of siderite. The gas product components corresponding to hydroxyl and carbonyl are H_2_O_(g)_, CO, and CO_2_, respectively, assigned by searching the standard spectral library. This shows that peaks I and IV in Figure 5a are the IR absorption bands of H_2_O_(g)_, peak II is the IR absorption band of CO_2_, and peak III is the IR absorption band of CO.

Based on the mineral and chemical composition of siderite, it was inferred that the following reactions occur during the magnetization roasting process [2,3,4,26].

FeCO_3_ → FeO + CO_2_↑ (7)

3FeO + CO_2_ → Fe_3_O_4_ + CO↑ (8)

KAl_2_(Si_3_Al)O_10_(OH)_2_ → KAl_2_(Si_3_Al)O_11_ + H_2_O_(g)_↑ (9)

The gaseous product released in the first stage is H_2_O_(g__)_, the mass loss of which could be a result of the removal of adsorbed water, according to the analysis shown in Figure 4a,b, and Figure 5a. The gaseous products released in the second stage are CO_2_ and H_2_O_(g)_. A clear CO_2_ release process can be observed on peak II in Figure 5a, and the IR absorption intensity of H_2_O_(g)_ also increases significantly. Based on the mineralogical and chemical composition of siderite, it was inferred that the release of CO_2_ is due to the decomposition of trace carbonates, while the release of H_2_O_(g)_ can be mainly attributed to the loss of crystalline water of muscovite. In the third stage, the IR absorption intensity of H_2_O_(g)_ shown in Figure 5a increases significantly and a strong CO_2_ release process occurs on peak II. This phase is mainly a decomposition process of siderite and white muscovite. The detection of CO confirms the formation of magnetite during the decomposition of FeCO_3_, consistent with the literature [10].

**Figure 6:**
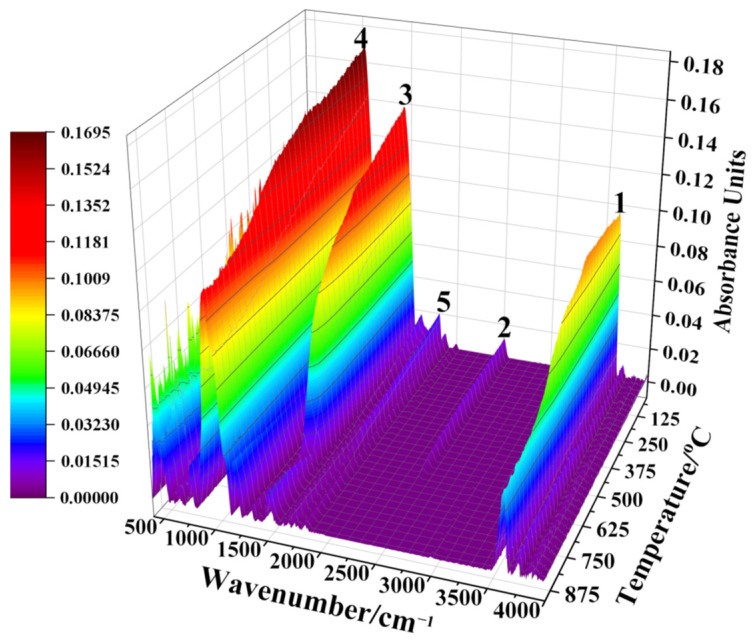
Online in-situ IR spectra of siderite. 1,2−IR absorption bands of hydroxyl groups; 3−IR absorption band of carbonate groups; 4−IR absorption band of silicates; 5−absorption bands of iron oxides.

The structural transformation of siderite samples in magnetization roasting can be directly characterized using online in-situ IR. The online in-situ 3D IR spectra of the magnetization roasting process of siderite at 15 °C/min are shown in Figure 6.

**Figure 7:**
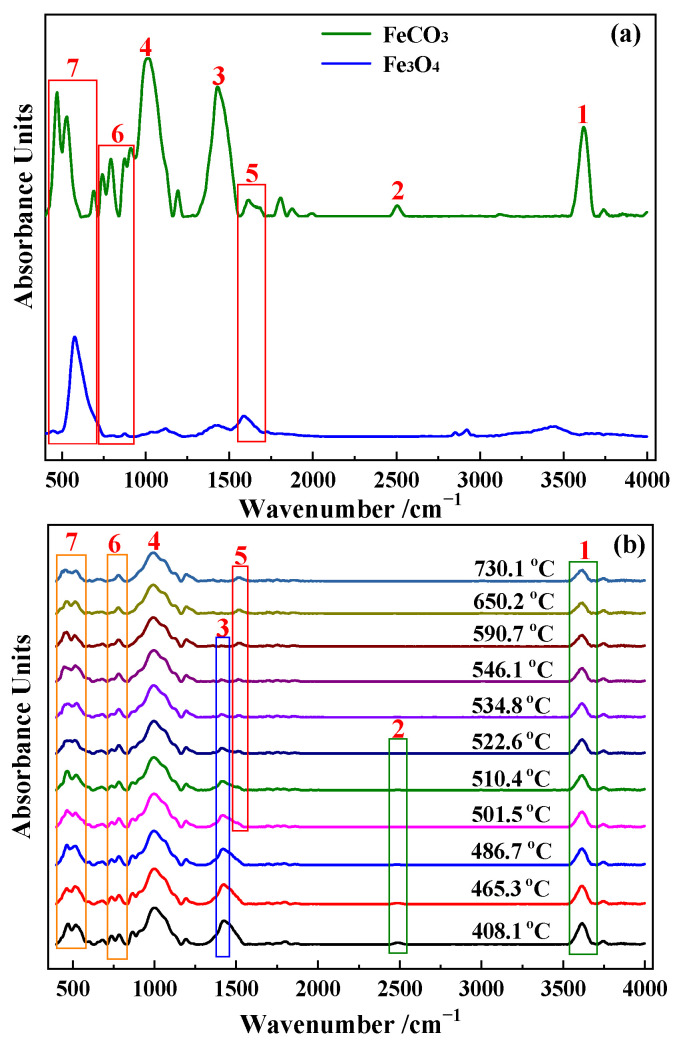
Two-dimensional IR of (**a**) magnetite–siderite and (**b**) roasted ore.

The composition of mineral materials is very complex, and trace amounts of minerals also produce complex IR absorption bands, therefore making the analysis of in-situ IR spectra very difficult. The most commonly used IR method is the determination of the standard spectra of pure substances, from which characteristic IR absorption bands are determined. The IR absorption spectrum of the pure substance is then used to characterize its structural changes. In this study, IR absorption spectra of a standard sample of magnetite and raw Daxigou siderite ore were measured, with the results shown in Figure 7a. The online in-situ IR absorption spectra of the roasted siderite ore are shown in Figure 7b, with the results in Figure 7a,b used to analyze the reaction characteristics of siderite in the magnetization roasting process.

The IR absorption spectrum of siderite is very complex, as shown in Figure 7a. According to the literature [27], in IR spectra, carbonates typically give rise to signals in the 1600–1300 cm^−1^ region, Si–O bond peaks are typically observed in the 1200–1100 cm^−1^ region, hydroxyl group peaks in the 3550–3200 cm^−1^ and 1100–1000 cm^−1^ regions, carbonate and silicate peaks in the 800–745 cm^−1^ region, and iron oxide and silicate peaks are observed in the 1700–1500 cm^−1^ and 705–400 cm^−1^. From this, it can be determined that peaks 1 and 2 in Figure 7a can be attributed to the IR absorption bands of hydroxyl groups (free water or water of crystallization); peak 3 is the IR absorption band of carbonate groups; peak 4 can be mainly attributed to the IR absorption band of silicates; and peaks 5, 6, and 7 can mainly be assigned as the absorption bands of iron oxides.

In the magnetization roasting of siderite, the decomposition of FeCO_3_ and the formation of magnetite are the key reactions. Figure 6 and Figure 7b show that the intensity of the carbonate absorption band marked as peak 3 decreases with a gradual increase in temperature. When the temperature reaches 590.7 °C, absorption band 3 almost completely disappears, indicating the completion of the FeCO_3_ decomposition, consistent with the TG-DTG results in Figure 4 and FTIR analysis in Figure 5. Peaks 1 and 2 are the absorption bands of hydroxyl groups, which are known to be related to muscovite based on the chemical composition of siderite. A closer observation of the data in Figure 6 shows that peak 1 is actually a merged absorption band with a smaller wavenumber at the top of the peak and a larger wavenumber at the bottom. With the gradual removal of hydroxyl groups, the upper part of the absorption band gradually weakens in intensity and disappears at around 570 °C, while the lower part shows no significant changes. Figure 6 shows that absorption band 2 disappears at around 570 °C, indicating the complete loss of crystal water from muscovite.

The IR absorption bands associated with the formation of magnetite are peaks 5, 6, and 7. As shown in Figure 7a, the peak 5 absorption band of siderite is a double peak, while the absorption band of magnetite is a single peak. Figure 6 shows that the bimodal peak of siderite gradually decreases with increasing temperature and disappears at around 500 °C, while a single peak that gradually increases in intensity starts to appear, indicating the gradual formation of magnetite. From peak 5 in Figure 7b, it can be clearly observed that the absorption band of magnetite starts to form from around 501 °C and gradually increases in intensity. After 590 °C, no further significant changes occur, indicating the completion of the magnetization reaction. The conversion process of siderite to magnetite can also be observed in the absorption band marked as peak 7. The major change is the gradual weakening and broadening of the left peak in the bimodal absorption band of siderite, while the right peak is gradually raised and shifted in the direction of increasing wavenumber. Figure 7a shows that the weakening and broadening of the left peak can be attributed to the decomposition of siderite, while the more intense right peak and its migration to higher wavenumbers is evidence of the increase in the formation of magnetite. Online in-situ IR analysis results indicate that the decomposition of siderite is completed at approximately 590 °C. Magnetite is gradually produced during the decomposition of siderite, and the magnetization reaction is completed simultaneously at approximately 590 °C after the complete decomposition of siderite.

### 3.2. Kinetics Analysis

**Figure 8:**
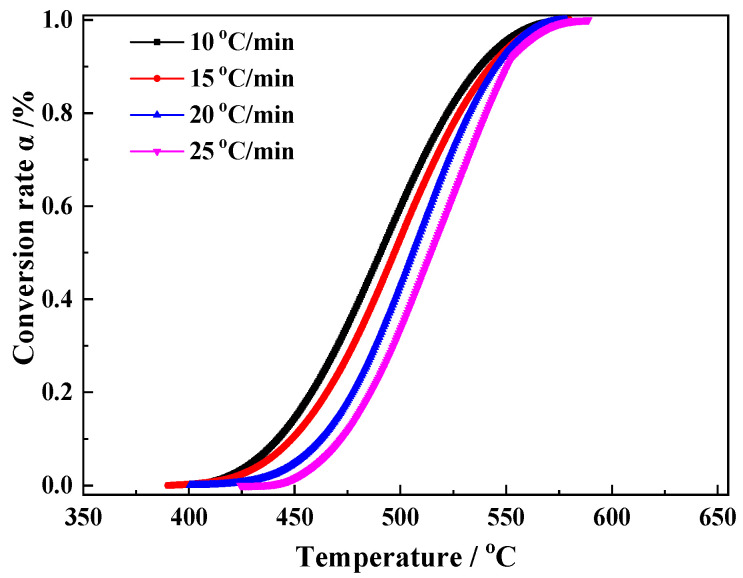
Conversion rate graph.

The key to the development of the integrated utilization technology of siderite is the kinetics characteristics of the magnetization reaction. Characterization of the magnetization reaction kinetics of siderite is difficult due to the lack of available real-time techniques for the measurement of iron minerals during magnetization roasting. However, the decomposition of FeCO_3_ in siderite and the formation of magnetite are almost synchronous, so the TG data of FeCO_3_ decomposition can be used in kinetic calculations. The reaction conversion *α* can be calculated from the TG data at four heating rates of 10 °C/min, 15 °C/min, 20 °C/min, and 25 °C/min, according to Equation (3), and the graph of *α* − *T* is plotted in Figure 8.

**Figure 9:**
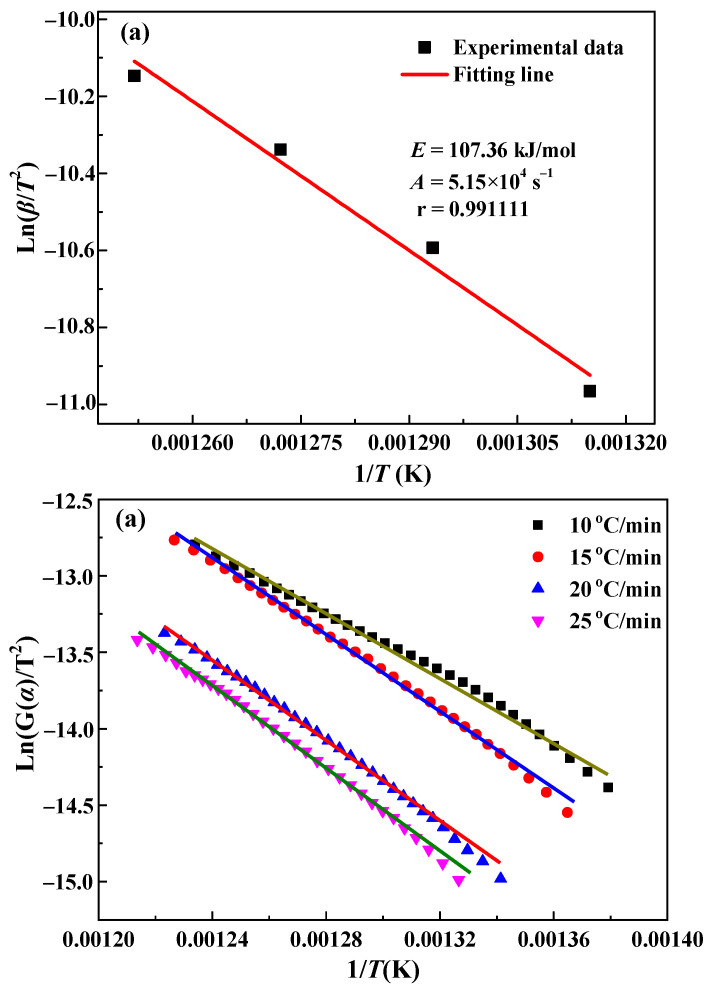
Kinetic fitting of the (**a**) Kissinger and (**b**) general integration methods.

Reaction kinetics were calculated using the Kissinger and general integral methods. The Kissinger method adopts the peak temperature labeled in Figure 4b for kinetic calculations according to Equation (4). The general integration method uses the *α* − *T* data shown in Figure 7 to perform a linear fit according to Equation (5), thereby allowing the calculation of the kinetic parameters E, A, and G(*α*). The 41 G(*α*) functions listed in the literature were fitted and calculated sequentially [23]. The results show that the highest linearity is observed for mechanism function No. 15. The plots fitted using the Kissinger and general integral methods using function No. 15 are shown in Figure 9a,b, respectively.

The fitting results shown in Figure 9 are listed in full in Table 2.

As shown in Table 2, the linear correlation coefficients, *r*, for both the Kissinger and general integration methods are >0.99. The relative deviation of the activation energy of the two methods is only 1.23%, indicating that the fitting results are reasonable. The mechanism of mechanism function No. 15 is random nucleation and growth, indicating that the magnetization roasting reaction of siderite is controlled by the nucleation and growth rate of magnetite [23]. The expression of mechanism function No. 15 is as follows.

(10) $$G\left(\alpha \right)={\left[-Ln\left(1-\alpha \right)\right]}^{\frac{3}{4}}$$

The kinetic equation for the magnetization roasting reaction of siderite is obtained by taking the average value of the general integration method as the final result, as follows:

(11) $${\left[-Ln\left(1-\alpha \right)\right]}^{\frac{3}{4}}=3.51\times 10^{5}exp\left(-\frac{106.1}{RT}\right)t$$

The time required for complete magnetization of siderite at different temperatures can be predicted using the kinetic equations, thus providing basic parameters for process design and reaction control. Table 3 shows the predicted results of the time required for the magnetization of siderite in the range of 600–780 °C using the kinetic Equation (11).

The kinetics predictions show that the times required to complete the magnetization of the siderite are 27.02 s, 12.24 s, 6.02 s, 3.17 s, and 2.22 s for calcination at 600 °C, 650 °C, 700 °C, 750 °C, and 780 °C, respectively. At 700–780 °C, it only took a few seconds to complete the magnetization of siderite, providing a basis for the design of the conveyor bed magnetization roasting reactor.

### 3.3. Characterization of Roasted Ore

#### 3.3.1. Magnetic Conversion Analysis

A conveyor bed roasting pilot test system was used for the magnetization roasting of siderite. over a temperature range of 600–780 °C, with an effective residence time of the material in the suspension reactor of around 3.5 s. A total of five temperatures were set for the pilot run of 72 h, with each temperature used for 12 h. Samples were taken every 2 h, with a total of six samples collected at each temperature. The average values of the analytical results of the iron minerals and magnetic conversion of the six roasted ores are listed in Table 4.

As shown in Table 4, the decomposition of FeCO_3_ in siderite was completed at roasting temperatures of 750 °C and 780 °C, with magnetic conversions of >0.99 within an effective residence time of approximately 3.5 s. The experimental results confirm that the conveyor bed process system is effective for the application of the magnetized roasting of siderite. The use of a three-stage suspension cooling process enables rapid cooling of the roasted ore to <200 °C and prevents the oxidation of magnetite [28,29,30]. The low hematite (magnetite) content of the roasted ore confirmed that the cooling process was effective. Compared to the ore roasted at 780 °C, ore roasted at 750 °C exhibits the highest magnetite grade and is calcined at a lower temperature, representing more optimal conditions.

**Figure 10:**
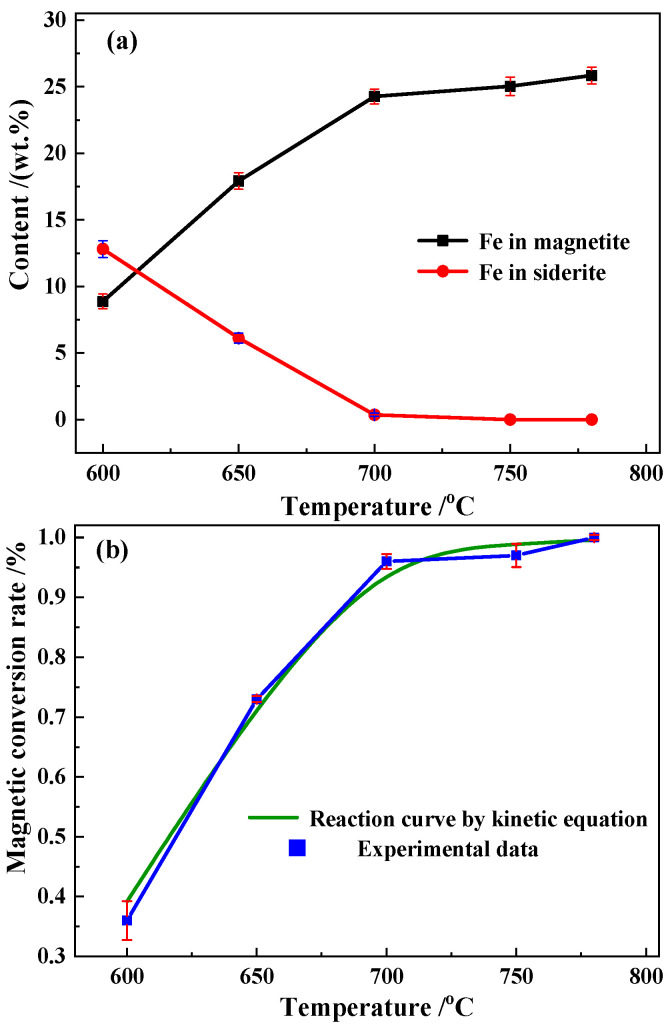
(**a**) Magnetite and (**b**) magnetic conversion curves.

The changes in FeCO_3_ and magnetite during the roasting of siderite are shown in Figure 10a. The experimental data of magnetic conversion compared with the predicted kinetics results are shown in Figure 10b.

Figure 10b shows that the kinetically predicted magnetic conversion curves are in agreement with the trends in the experimental data. The deviation of the data was <3.8%, indicating that the kinetic model was reasonable. It was thus, feasible to use the kinetic equations for the parameterized design and production control of the conveyor bed process system.

#### 3.3.2. XRD Analysis

**Figure 11:**
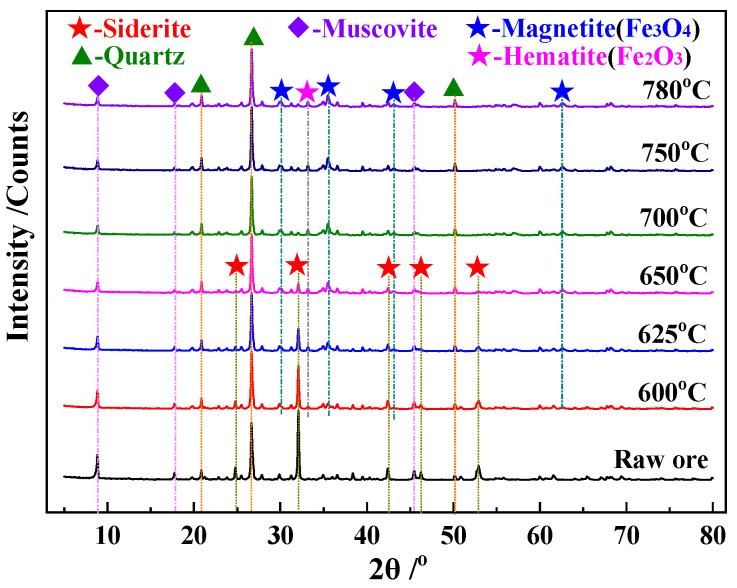
XRD patterns of the roasted ore recorded at different temperatures.

XRD patterns of the roasted ore are shown in Figure 11.

As shown in Figure 11, the FeCO_3_ in siderite decomposes almost completely at 700 °C, and magnetite starts to be produced in large quantities at 650 °C, with its XRD peak intensity remaining almost unchanged >700 °C. Overall, the results of the XRD analysis are in general agreement with the data presented in Table 4.

#### 3.3.3. BSE-EDS Analysis

**Figure 12:**
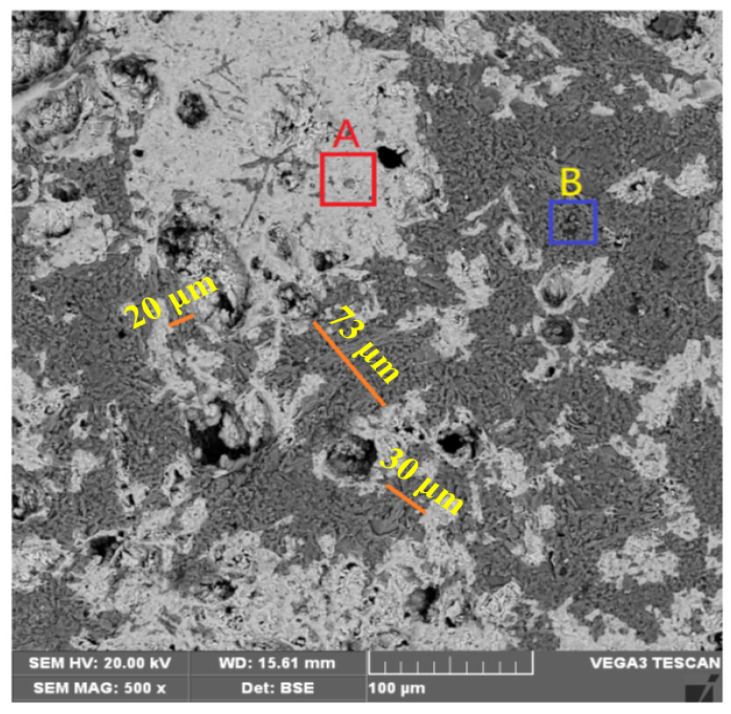
BSE image of the roasted ore.

A BSE image of the magnetized ore roasted at 750 °C is shown in Figure 12. The results of the EDS analysis of regions A and B are shown in Table 5.

According to the EDS analysis of Figure 12, region A is dominated by magnetite, with region B dominated by quartz and muscovite. The distribution state of the magnetite and veinstone minerals is more complex. Magnetite distributed in large continuous masses can be well dissociated on a scale of 50–80 μm. However, for magnetite distributed as microfine particles interspersed with veinlets, dissociation needs to occur on a scale of 20–30 μm, therefore necessitating ultra-fine grinding.

### 3.4. Magnetic Separation Analysis

The roasted ore produced by the conveyor bed magnetization roasting system at 750 °C was magnetically sorted according to the beneficiation process shown in Figure 3. The products from the beneficiation process were analyzed in terms of their iron grade, recovery, and yield, with the results shown in Table 6.

The final iron grade of the concentrate obtained after the multi-stage magnetic separation process was 62.80 wt.% with a recovery of 68.83%, which meets the requirements of steel smelting, as shown in Table 6. The middling ore III and IV obtained in the beneficiation process can be re-added into the beneficiation process to improve the overall recovery in future applications. The conveyor bed magnetization roasting and magnetic separation processes were used to recover 68.83% Fe from Daxigou siderite ore. The processes are also suitable for the upgrading and utilization of low-grade siderite.

## 4. Conclusions

(1) Magnetite was prepared by roasting siderite under an inert and reducing atmosphere, which was almost synchronous with the decomposition of siderite. The reaction mechanism of the magnetization roasting of magnetite was determined to be random nucleation and subsequent growth, with a reaction activation energy of 106.1 kJ/mol.
(2) Roasted ore with a magnetic conversion of ≥0.99 was obtained using a conveyor bed system at 750 °C and 780 °C for around 3.5 s. Magnetite oxidation was prevented via the adoption of a three-stage suspended state air cooling process. The kinetic model predictions were consistent with the experimental results.
(3) The conveyor bed magnetization roasting and magnetic separation process were suitable for the upgrading and utilization of low-grade iron ore. For the iron grade of Daxigou siderite with 21.42 wt.% original ore, a concentrate with an iron grade of 62.80 wt.% and a recovery rate of 68.83% was obtained after conducting the conveyor bed magnetization roasting and magnetic separation process.

## Figures and Tables

**Table 1:** Chemical composition of siderite /(wt.%).

Al_2_O_3_	SiO_2_	Fe_2_O_3_	CaO	MgO	MnO	K_2_O	SO_3_	Mass loss	T_Fe_
11.18	36.82	40.12	0.81	1.95	0.69	2.60	0.38	11.63	21.42

**Table 2:** Results of the fitting of the reaction kinetics.

Method	*β*/(°C/min)	*E*/(kJ/mol)	*A*	*r*
General integral method	10	98.1	7.58 × 10^4^	0.995680
	15	103.4	2.35 × 10^5^	0.995052
	20	108.8	3.58 × 10^5^	0.995351
	30	114.0	7.37 × 10^5^	0.997411
	Average	106.1	3.51 × 10^5^	0.995874
Kissinger method	-	107.4	5.15 × 10^5^	0.991111

**Table 3:** Prediction of the time required for siderite magnetization using the kinetic equations.

Temperature /(°C)	*α* = 0.80	*α* = 0.90	*α* = 0.95	*α* = 0.98	*α* = 0.999
600	9.06	11.85	14.44	17.64	27.02
650	4.11	5.37	6.54	7.99	12.24
700	2.02	2.64	3.22	3.93	6.02
750	1.06	1.39	1.69	2.07	3.17
780	0.75	0.97	1.19	1.45	2.22

**Table 4:** Results of the analysis of the iron minerals and magnetic conversion of roasted ore.

Temperature (°C)	Fe in Magnetite (wt.%)	Fe in Siderite (wt.%)	Fe in Hematite (wt.%)	Fe in Silicate (wt.%)	Fe in Sulfide (wt.%)	Total (wt.%)	Standard Deviation	Magnetic Conversion Rate (%)
Raw ore	0.21	19.58	0.84	0.56	0.23	21.42	0.0299	0.0
600	8.87	12.81	0.76	0.59	0.18	23.21	0.0294	0.39
650	17.92	6.12	0.56	0.63	0.12	25.35	0.0258	0.72
700	24.27	0.35	0.32	0.64	0.02	25.60	0.0275	0.97
750	25.83	0	0.27	0.64	0	26.74	0.0321	0.99
780	25.75	0	0.11	0.62	0	26.48	0.0327	1.00

**Table 5:** EDS analysis of regions A and B (in Figure 12).

Region A			Region B		
Element	Mass Percentage (%)	Atomic Percentage (%)	Element	Mass Percentage (%)	Atomic Percentage (%)
O	26.81	49.52	O	43.06	57.93
Fe	62.6	32.84	Si	48.25	36.97
Si	5.15	5.42	Fe	3.23	1.24
Mg	0.87	1.05	K	2.03	1.12
Ca	0.30	0.22	Al	3.43	2.74

**Table 6:** Analysis results of the iron grade, recovery, and yield.

Product Type	Yield (%)	Iron Grade (wt.%)	Iron Recovery (%)
Concentrate	28.79	62.80	68.83
Middling IV	2.76	58.04	6.10
Middling III	2.22	44.70	3.78
Middling II	13.90	12.82	6.78
Middling I	22.92	12.97	11.32
Tailings	29.41	2.85	3.19
Roasted ore	100.00	26.32	100

Note: the data in Table 6 are the averages of parallel samples.

## Data Availability

The data used to support the findings of this study are available from the corresponding author upon request.
